# Effects of odor on emotion, with implications

**DOI:** 10.3389/fnsys.2013.00066

**Published:** 2013-10-10

**Authors:** Mikiko Kadohisa

**Affiliations:** MRC Cognition and Brain Sciences Unit, Department of Experimental Psychology, University of OxfordOxford, UK

**Keywords:** odor, emotion, amygdala, hippocampus, prefrontal cortex

## Abstract

The sense of smell is found widely in the animal kingdom. Human and animal studies show that odor perception is modulated by experience and/or physiological state (such as hunger), and that some odors can arouse emotion, and can lead to the recall of emotional memories. Further, odors can influence psychological and physiological states. Individual odorants are mapped via gene-specified receptors to corresponding glomeruli in the olfactory bulb, which directly projects to the piriform cortex and the amygdala without a thalamic relay. The odors to which a glomerulus responds reflect the chemical structure of the odorant. The piriform cortex and the amygdala both project to the orbitofrontal cortex (OFC) which with the amygdala is involved in emotion and associative learning, and to the entorhinal/hippocampal system which is involved in long-term memory including episodic memory. Evidence that some odors can modulate emotion and cognition is described, and the possible implications for the treatment of psychological problems, for example in reducing the effects of stress, are considered.

## Introduction

Inputs received through the sensory systems, individually or together, can produce emotion and influence cognition (LeDoux, 1998; Rolls, 2005; Adolphs, 2010). Emotions can be elicited by stimuli that are instrumental reinforcers (Rolls, 2005). For example, the sight of an impending painful stimulus produces the emotional state of fear, and instrumental actions are performed to avoid the stimulus. Emotional states are generally associated with autonomic responses such as a change in heart rate, and endocrine responses, which are adaptive in that they prepare the body for action. Different reinforcing stimuli arouse different emotional states. Rewarding stimuli are associated with pleasant subjective states, and punishing stimuli with unpleasant subjective states. Human imaging studies have shown that reinforcing visual, auditory, olfactory and taste stimuli activate brain areas such as the orbitofrontal cortex (OFC), and that the activations are linearly correlated with the subjectively reported pleasantness or unpleasantness of the stimuli (Phan et al., 2002; Anderson et al., 2003; Kringelbach et al., 2003; Zald, 2003; Grabenhorst et al., 2007; Anders et al., 2008; Rolls et al., 2008). Odors may be particularly effective stimuli for the recall of memories including emotional memories (Herz, 1996).

The present review addresses two specific questions. First, what is the evidence for a strong link between odors and emotion? Second, what aspects of olfaction and the olfactory system predispose it to produce emotional responses? I begin by reviewing current definitions of emotion, and current views about the neurobiology of odor perception. The unique neuroanatomy and corresponding evolutionary history of the mammalian olfactory system (Lledo et al., 2005) suggest that it could be a very effective, often sub-conscious, driver of emotional responses. Further, the use of pleasant olfactory stimuli, acting as safety signals or reminders of a safe or pleasant event or place, may be useful in the treatment of psychiatric and related conditions, for example reducing anxiety (Lehrner et al., 2000, 2005).

## What is emotion?

Darwin described expressions and gestures that are associated with emotion in humans and other animals (Darwin, 1872). Emotions are also associated with adaptive changes of physiological state including heart rate and endocrine responses (Darwin, 1872; LeDoux, 1998; Rolls, 2005; Adolphs, 2010; LeDoux, 2012). Emotions can usefully be defined as states elicited by stimuli that are rewards or punishers, that is which have value (Rolls, 2005). For example, the “fear” evoked by the smell of a predator is associated with actions to avoid the predator, changes in heart-rate, increased blood flow directed to skeletal muscles, adrenaline release, etc. that maximize the ability to expend energy to escape. Humans might report that this constellation of adaptive autonomic, endocrine, and behavioral responses is associated with an introspective or conscious label of “fear”. Although Ekman suggested that there is a set of basic emotions, fear, anger, enjoyment, sadness, disgust, and surprise (Ekman, 1992), a much larger range of emotions can be accounted for if we take into account the rewarding or punishing effects of many different types of reinforcer, such as the taste of food, pleasant touch or pain, and the sight of a beautiful person or scene. Humans and non-human animals alike will perform actions to obtain a stimulus which is a reward, and will perform actions to avoid or escape from a punisher, and these reinforced actions are fundamental to survival and reproductive success (Rolls, 2005).

## Perception of odor

Odor perception can be influenced by learning and memory (Wilson and Stevenson, 2003a,b, 2006), for example exposure to wine or beer significantly improves discrimination (Owen and Machamer, 1979; Peron and Allen, 1988).

The piriform cortex plays a role in odor perception by recoding odor mixtures in such a way that some neurons can respond to an odorant mixture but not to the components (Kadohisa and Wilson, 2006b; Wilson and Sullivan, 2011). In particular, the anterior piriform cortex (aPC) encodes the identity of mixtures of odorants separately from the components, while the posterior piriform cortex (pPC) encodes the similarity between mixtures and their components (Gottfried et al., 2006; Kadohisa and Wilson, 2006b; Wilson and Sullivan, 2011). The implication is that the aPC enables complex mixtures to be represented separately from their components and from each other, while the pPC may enable generalization to similar odors. Another aspect of odor perception is short or long term habituation. aPC neurons adapt to a repeated or prolonged odor rapidly (Wilson, 1998; Wilson and Linster, 2008), which facilitates segmentation of an odor from the background odor, that is, aPC neurons detect a new odor against an adapted background odor (Kadohisa and Wilson, 2006a). This neurophysiological adaptation may be useful in drawing attention to harmful chemicals in adapted background odor environments.

Physiological state influences the perception of odor. For example, the odor of a food will be pleasant during hunger, and its pleasantness will decline as the food is being eaten to satiety (Rolls and Rolls, 1997; Yeshurun and Sobel, 2010). This can be a sensory-specific decrease in the pleasantness of the odor of a food eaten to satiety. In addition, the perception of odors can lead to physiological responses, such as salivation and the release of insulin (Lee and Linden, 1992; Yeomans, 2006; Savigner et al., 2009). Thus physiological state and the effects of odors can interact to influence motivation, for example whether to eat or not. In addition, odors influence autonomic responses, for example pleasant and novel odors can decrease heart rate (Fletcher and Wilson, 2001; Wilson, 2009), while the more sympathetically arousing the odor, the more the skin conductance response increases (Alaoui-Ismaili et al., 1997; Bensafi et al., 2002).

Thus, odor perception is neurophysiologically modulated by experience to facilitate identification of individual odorants including odor mixtures, and to filter out an odor by adaptation, and is also modified by physiological states such as hunger.

## Olfactory processing streams in the brain

There are two different olfactory systems. So far, I have considered the main olfactory epithelium (MOE), which projects to the main olfactory bulb (MOB). In addition, there is an accessory olfactory system with its peripheral receptors located in the vomeronasal organ (VNO) which projects to the accessory olfactory bulb (AOB). The VNO contributes to the detection of pheromones while the MOB detects volatile odors including pheromones. The VNO of mice detects opposite-sex urinary volatiles, in contrast to the MOB which detects volatiles from both sexes (Martel and Baum, 2007; Baum, 2012). The VNOs of humans and old world monkeys are non-functional (Liman and Innan, 2003; Zhang and Webb, 2003; Brennan and Zufall, 2006) although pheromones play important roles in eliciting social and reproductive behavior. In humans, the MOB may be responsible for pheromone detection (Preti et al., 2003; Wysocki and Preti, 2004).

Considering the main olfactory system, the glomerular layer of the MOB forms odorant receptor maps which reflect individual odorant molecular features (Mori et al., 2006) and the behavioral effects such as escape that they produce (Kobayakawa et al., 2007; Mainen, 2007; Matsumoto et al., 2010; Mori and Sakano, 2011).

Odor information reaches the piriform cortex, amygdala, and entorhinal cortex from the MOB. As shown above, the aPC separates representations of odor mixtures from their components. The piriform cortex projects to the amygdala and prefrontal cortex (PFC) (OFC in primates) (Barbas and De Olmos, 1990; Carmichael et al., 1994; Carmichael and Price, 1995; Pitkanen et al., 2000). The amygdala and PFC are involved in the processes of emotion including emotion-related learning (Zald and Pardo, 1997; Royet et al., 2003; Rolls, 2004) as described below. The MOB and piriform cortex also project to the entorhinal cortex, which in turn projects to the hippocampus where olfactory information can become incorporated into episodic long-term memory (Rolls, 2010; Small et al., 2011; Olsen et al., 2012) as described below.

*Amygdala*: The MOB projects directly to the anterior cortical nucleus and medial nucleus of amygdala, and the periamygdaloid cortex in rodents and macaques (Turner et al., 1978; Turner and Mishkin, 1978; Carmichael et al., 1994; McDonald, 1998), while the AOB projects to the medial nucleus of the amygdala in rodents (Keverne, 1999; Pitkanen, 2000; Kang et al., 2009). The medial nucleus of the amygdala is thought to be a place for inputs from the AOB and MOB of rodents to interact with each other (Brennan and Zufall, 2006; Baum, 2012). In addition, the piriform cortex projects directly to the basolateral nucleus of amygdala in both rodents and macaques (Carmichael et al., 1994; McDonald, 1998). Further, the amygdala projects to the hypothalamus (Pitkanen, 2000; Barbas et al., 2003; Gabbott et al., 2012), which may provide a route for olfactory stimuli to influence the autonomic and endocrine systems.

The amygdala plays a key role in some affective responses to stimuli. Amygdala-lesioned animals show a lack of reactions such as freezing, and autonomic responses, to a fear conditioned stimulus (CS; Weiskrantz, 1956; Helmstetter, 1992; Phillips and LeDoux, 1992; Sananes and Davis, 1992), and show abnormal food preferences including reduced neophobia (Rolls and Rolls, 1973; Murray et al., 1996). In addition, the amygdala plays a role in associative learning, for example, some amygdala neurons encode odor cues associated with a positive or negative taste (Schoenbaum et al., 1999).

*Hippocampus*: The hippocampus receives olfactory information from the amygdala (Petrovich et al., 2001) and from the entorhinal cortex which receives inputs from the MOB and piriform cortex (Price, 1973; Witter and Amaral, 1991; Carmichael et al., 1994).

The hippocampus plays a fundamental role in episodic memory (Lemogne et al., 2006; Rolls, 2010; Small et al., 2011; Olsen et al., 2012). Hippocampal damage impairs odor-place associative learning, and temporal order memory for odor information (Kesner et al., 2002; Rolls and Kesner, 2006). Some neurons in the hippocampus represent the place of odor-related reward (Rolls, 2010; Tort et al., 2011). In addition, stimulation of the hippocampus affects autonomic responses, that is, decreases heart rate and blood pressure (Ruit and Neafsey, 1988).

*Prefrontal cortex*: The primate OFC receives olfactory information from the piriform cortex (Carmichael and Price, 1994), the amygdala (Amaral and Price, 1984; Ghashghaei and Barbas, 2002; Barbas et al., 2003), parahippocampal cortices (Carmichael and Price, 1995), and from the hypothalamus (Tanabe et al., 1975; Barbas et al., 2003).

The OFC associates olfactory with taste, oral texture, and visual inputs, to produce multimodal representations of reward value, such as the reward value of food (Rolls, 2012). The OFC representations are of the economic value of goods, e.g., of a food (Padoa-Schioppa, 2011). The reward evaluation performed by the OFC is important in both emotion and in autonomic responses such as heart rate and skin conductance responses (Damasio, 1995; Bechara et al., 1996).

In addition, the amygdala, OFC, and hippocampus are also involved in the regulation of stress which causes not only psychological and psychiatric but also physiological problems (see Mcewen, 2007; Arnsten, 2009; Rodrigues et al., 2009; Ulrich-Lai and Herman, 2009). As described below, the olfactory pathways may enable some odorants to influence stress in a therapeutic way.

## Odor-evoked emotion and odor-associated emotional memory

There is evidence that odors elicit emotion and are linked to emotional memory. Responses to odors can include emotions, which can usefully be defined as states elicited by stimuli that are rewards and punishers (Rolls, 2005). For examples, a predator’s odor can cause a prey to escape from it, while newborn babies move towards the breast odor of their own mother (Varendi and Porter, 2001). Different odors can elicit individual affective responses which may be pleasant or unpleasant (Chrea et al., 2009; Seubert et al., 2009, 2010). Emotionally arousing odors may influence cognition and emotion. Some odors facilitate the recognition of disgust face expressions (Seubert et al., 2010) and impair working memory (Schneider et al., 2006; Habel et al., 2007) while others reduce anger (Rétiveau et al., 2004) or improve mood (Schiffman et al., 1995). In addition, some affective odors modulate psychological and physiological state. Komori and colleagues (Komori et al., 1995) reported effects of citrus fragrance on the improvement of psychological states and immune function of patients with depression. Green odor emanating from oak leaves is necessary for polyphemus moths to mate (Riddiford, 1967), and elicits pleasantness from humans (Sano et al., 2002). In addition, green odor attenuates the stress-induced elevations of plasma adrenocorticotropic hormone (ACTH), body temperature (Nakashima et al., 2004), and skin-barrier disruption in rats (Fukada et al., 2007). Similarly, rose essential oil inhibits stress-induced skin-barrier disruption and elevation of salivary cortisol in humans (Fukada et al., 2012), and orange and lavender reduce the anxiety of patients in a dental office (Lehrner et al., 2000, 2005).

Odors can become associated by learning with reinforcers such as taste in the OFC and amygdala, and this provides a way for previously neutral odors to produce emotional responses (Herz et al., 2004; Rolls, 2005). Patients frightened by dental treatment respond negatively to the smell of eugenol which is used for treatment (Robin et al., 1998). Odor-evoked autobiographical memory is powerful in reminding humans about past emotional experiences (Chu and Downes, 2002; Larsson and Willander, 2009; Zucco et al., 2012). This odor associative memory and learning begins early in life (Schaal et al., 2000; Larsson and Willander, 2009). For examples, the foods eaten by a pregnant mother can influence the odors liked by her offspring (Schaal et al., 2000), and neonatal rats at ages of less than 3 days learn the odor associated with aversive or rewarded outcomes (Rudy and Cheatle, 1977; Johanson and Hall, 1979). Further, this associative learning regulates behavior, that is, when animals have learned associations between an odor CS and a negative outcome such as foot shock unconditioned stimulus (US), they try to avoid the CS associated with the negative US, or show fear expressions such as freezing (Sullivan et al., 2000; Sacco and Sacchetti, 2010).

The amygdala, OFC and hippocampus are involved in the process of odor-elicited emotion and odor-associated emotional memory. Human imaging studies have demonstrated that the amygdala and OFC are activated by unpleasant or pleasant odors, consistent with their functions in emotion (Zald and Pardo, 1997; Royet et al., 2003; Small et al., 2003). Consistently, activations of the human OFC elicited by odors correlate with the subjective pleasantness or unpleasantness of the odors (Rolls, 2000; Anderson et al., 2003; Grabenhorst et al., 2007). Amygdala lesions impair learned odor preferences in infant rats (Sullivan and Wilson, 1993) while hippocampus and subiculum lesions in rats produce a deficit in social learning of an odor association (Alvarez et al., 2002). In humans, the amygdala and PFC of patients with posttraumatic stress disorder (PTSD) are activated by exposure to the odor related to the traumatic memory (Vermetten et al., 2007) while the hippocampus and PFC are activated by odor cues during autobiographical memory retrieval process (Larsson and Willander, 2009).

The odor mapping performed in the aPC may set up representations of odors that are separate, even when considering odorant mixtures and their components (Kadohisa and Wilson, 2006b; Wilson and Sullivan, 2011). This pattern separation of the representations of even odorant mixtures using non-linear remapping is useful for the pattern association learning of odors to taste and visual representations performed in the OFC and amygdala to represent the reward/emotional value of the odor object (Rolls, 2008), and for the autoassociative formation of episodic memory performed in the hippocampus (Rolls, 2010).

## Conclusions

Human and complementary studies in non-human animals provide evidence that odors evoke emotion and autonomic state via pathways to the amygdala and OFC, and become incorporated into episodic memory via the hippocampus.

In addition, it is suggested that some odorants which elicit emotion may have potential to treat patients with psychological problem such as depression.

## Conflict of interest statement

The authors declare that the research was conducted in the absence of any commercial or financial relationships that could be construed as a potential conflict of interest.
